# (3*S*,4*Z*)-3-Chloro-1-methyl-4-[(2*E*)-(3-methyl­benzyl­idene)hydrazinyl­idene]-3,4-dihydro-1*H*-2,1-benzothia­zine 2,2-dioxide

**DOI:** 10.1107/S1600536811056315

**Published:** 2012-01-11

**Authors:** Muhammad Shafiq, M. Nawaz Tahir, Islam Ullah Khan, Muhammad Zia-Ur-Rehman

**Affiliations:** a Materials Chemistry Laboratory, Department of Chemistry, Government College University, Lahore, Pakistan; b University of Sargodha, Department of Physics, Sargodha, Pakistan; c Applied Chemistry Research Center, PCSIR Laboratories Complex, Lahore, Pakistan

## Abstract

In the title compound, C_17_H_16_ClN_3_O_2_S, the dihedral angle between the benzene rings is 7.75 (13)°. The thia­zine ring adopts an envelope conformation with the S atom as the flap at a distance of 0.813 (2) Å from the plane through the other five atoms. In the crystal, C—H⋯O hydrogen bonds link the mol­ecules into chains propagating in [100].

## Related literature

For related structures, see: Shafiq *et al.* (2011*a* ▶,*b* ▶,*c* ▶). For further synthetic details, see: Shafiq *et al.* (2011*d* ▶). For puckering parameters, see: Cremer & Pople (1975 ▶).
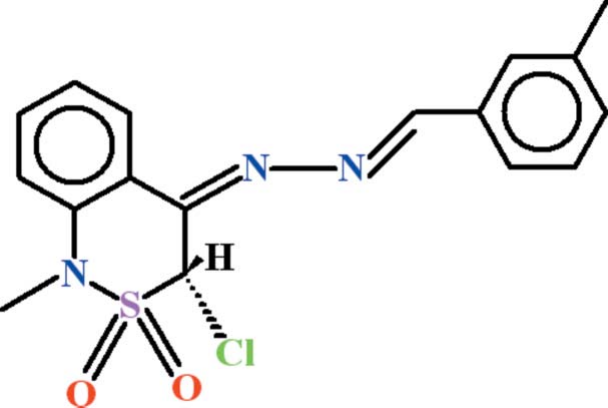


## Experimental

### 

#### Crystal data

C_17_H_16_ClN_3_O_2_S
*M* *_r_* = 361.84
Orthorhombic, 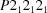

*a* = 8.7734 (2) Å
*b* = 11.1271 (2) Å
*c* = 17.9423 (3) Å
*V* = 1751.57 (6) Å^3^
*Z* = 4
Mo *K*α radiation
μ = 0.35 mm^−1^
*T* = 296 K
0.26 × 0.18 × 0.12 mm

#### Data collection

Bruker Kappa APEXII CCD diffractometer
Absorption correction: multi-scan (*SADABS*; Bruker, 2005 ▶) *T*_min_ = 0.930, *T*_max_ = 0.960
17152 measured reflections
4265 independent reflections
3478 reflections with *I* > 2σ(*I*)
*R*_int_ = 0.029

#### Refinement

*R*[*F* ^2^ > 2σ(*F* ^2^)] = 0.036
*wR*(*F* ^2^) = 0.088
*S* = 1.03
4265 reflections
219 parameters
H-atom parameters constrained
Δρ_max_ = 0.24 e Å^−3^
Δρ_min_ = −0.27 e Å^−3^
Absolute structure: Flack (1983 ▶), 1788 Friedel pairs
Flack parameter: 0.47 (6)

### 

Data collection: *APEX2* (Bruker, 2009 ▶); cell refinement: *SAINT* (Bruker, 2009 ▶); data reduction: *SAINT*; program(s) used to solve structure: *SHELXS97* (Sheldrick, 2008 ▶); program(s) used to refine structure: *SHELXL97* (Sheldrick, 2008 ▶); molecular graphics: *ORTEP-3 for Windows* (Farrugia, 1997 ▶) and *PLATON* (Spek, 2009 ▶); software used to prepare material for publication: *WinGX* (Farrugia, 1999 ▶) and *PLATON*.

## Supplementary Material

Crystal structure: contains datablock(s) global, I. DOI: 10.1107/S1600536811056315/hb6582sup1.cif

Structure factors: contains datablock(s) I. DOI: 10.1107/S1600536811056315/hb6582Isup2.hkl

Supplementary material file. DOI: 10.1107/S1600536811056315/hb6582Isup3.cml

Additional supplementary materials:  crystallographic information; 3D view; checkCIF report

## Figures and Tables

**Table 1:** Hydrogen-bond geometry (Å, °)

*D*—H⋯*A*	*D*—H	H⋯*A*	*D*⋯*A*	*D*—H⋯*A*
C4—H4⋯O2^i^	0.93	2.57	3.469 (3)	163
C10—H10⋯O1^ii^	0.93	2.53	3.300 (3)	140

Symmetry codes: (i) 
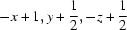
; (ii) 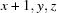.

**Fig. 1.**
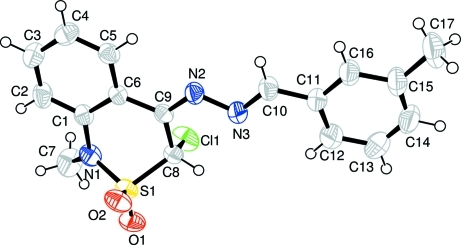
View of the title compound with displacement ellipsoids drawn at the 50% probability level.

**Fig. 2.**
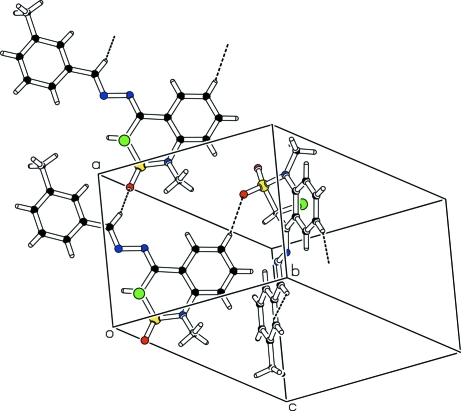
The partial packing showing chains extending along the [100] direction.

## supplementary crystallographic information

### Comment

The title compound (I), (Fig. 1) has been synthesized in continuation of our studies of Schiff bases (Shafiq *et al.*, 2011**a*,*b*,c*).

In (I), the benzene rings A (C1—C6) and B (C11—C16) are planar with r. m. s. deviation of 0.0033 and 0.0002 Å, respectively. The dihedral angle between A/B is 7.75 (13)°. The central group C (N2/N3/C10) is of course planar. The dihedral angle between A/C and B/C is 6.02 (19) and 5.11 (21)°, respectively. The thiazine ring D (C1/C6/C9/C8/S1/N1) is in the nvelope form, with the maximum puckering amplitude (Cremer & Pople, 1975), Q = 0.5707 (16) Å. The molecules form one-dimensional polymeric chains extending along the *a*-axis due to H-bonding of C—H···O type (Table 1).

### Experimental

Schiff base derivative of (4*Z*)-4-hydrazinylidene-1-methyl-3,4-dihydro -1*H*-2,1-benzothiazine 2,2-dioxide and 3-methylbenzaldehyde was prepared using the method reported previously (Shafiq *et al.* 2011*d*). The chlorination of the schiff base was undertaken using *N*-chloro succinimide and dibenzoylperoxide (Shafiq *et al.*, 2011*a*). The crude product was re-crystallized from ethyl acetate to yield orange needles of (I).

### Refinement

The H-atoms were positioned geometrically (C–H = 0.93–0.96 Å) and refined as riding with *U*_iso_(H) = x*U*_eq_(C), where x = 1.5 for methyl and x = 1.2 for aryl H-atoms.

### Crystal data

**Table:**

C_17_H_16_ClN_3_O_2_S	*F*(000) = 752
*M**_r_* = 361.84	*D*_x_ = 1.372 Mg m^−^^3^
Orthorhombic, *P*2_1_2_1_2_1_	Mo *K*α radiation, λ = 0.71073 Å
Hall symbol: P 2ac 2ab	Cell parameters from 2106 reflections
*a* = 8.7734 (2) Å	θ = 1.4–25.3°
*b* = 11.1271 (2) Å	µ = 0.35 mm^−^^1^
*c* = 17.9423 (3) Å	*T* = 296 K
*V* = 1751.57 (6) Å^3^	Needle, orange
*Z* = 4	0.26 × 0.18 × 0.12 mm

### Data collection

**Table:**

Bruker Kappa APEXII CCD diffractometer	4265 independent reflections
Radiation source: fine-focus sealed tube	3478 reflections with *I* > 2σ(*I*)
graphite	*R*_int_ = 0.029
Detector resolution: 7.50 pixels mm^-1^	θ_max_ = 28.3°, θ_min_ = 2.2°
ω scans	*h* = −10→11
Absorption correction: multi-scan (*SADABS*; Bruker, 2005)	*k* = −14→14
*T*_min_ = 0.930, *T*_max_ = 0.960	*l* = −23→23
17152 measured reflections	

### Refinement

**Table:**

Refinement on *F*^2^	Secondary atom site location: difference Fourier map
Least-squares matrix: full	Hydrogen site location: inferred from neighbouring sites
*R*[*F*^2^ > 2σ(*F*^2^)] = 0.036	H-atom parameters constrained
*wR*(*F*^2^) = 0.088	*w* = 1/[σ^2^(*F*_o_^2^) + (0.041*P*)^2^ + 0.2645*P*] where *P* = (*F*_o_^2^ + 2*F*_c_^2^)/3
*S* = 1.02	(Δ/σ)_max_ < 0.001
4265 reflections	Δρ_max_ = 0.24 e Å^−^^3^
219 parameters	Δρ_min_ = −0.26 e Å^−^^3^
0 restraints	Absolute structure: Flack (1983), 1788 Friedel pairs
Primary atom site location: structure-invariant direct methods	Flack parameter: 0.47 (6)

### Special details

**Table:**

Geometry. Bond distances, angles *etc*. have been calculated using the rounded fractional coordinates. All su's are estimated from the variances of the (full) variance-covariance matrix. The cell e.s.d.'s are taken into account in the estimation of distances, angles and torsion angles
Refinement. Refinement of *F*^2^ against ALL reflections. The weighted *R*-factor *wR* and goodness of fit *S* are based on *F*^2^, conventional *R*-factors *R* are based on *F*, with *F* set to zero for negative *F*^2^. The threshold expression of *F*^2^ > σ(*F*^2^) is used only for calculating *R*-factors(gt) *etc*. and is not relevant to the choice of reflections for refinement. *R*-factors based on *F*^2^ are statistically about twice as large as those based on *F*, and *R*- factors based on ALL data will be even larger.

### Fractional atomic coordinates and isotropic or equivalent isotropic displacement parameters (Å^2^)

**Table:**

	*x*	*y*	*z*	*U*_iso_*/*U*_eq_	
Cl1	0.16886 (7)	0.16151 (6)	0.00310 (3)	0.0619 (2)	
S1	0.10071 (6)	0.09643 (5)	0.15565 (3)	0.0411 (2)	
O1	−0.04053 (18)	0.05118 (15)	0.12847 (9)	0.0559 (6)	
O2	0.17040 (19)	0.04041 (14)	0.21814 (8)	0.0524 (5)	
N1	0.0827 (2)	0.23974 (17)	0.16940 (11)	0.0453 (6)	
N2	0.5175 (2)	0.13115 (15)	0.09335 (10)	0.0410 (5)	
N3	0.5229 (2)	0.03374 (16)	0.04310 (10)	0.0467 (6)	
C1	0.2182 (3)	0.30519 (17)	0.18673 (11)	0.0381 (6)	
C2	0.2065 (3)	0.4082 (2)	0.22991 (13)	0.0523 (8)	
C3	0.3334 (3)	0.4739 (2)	0.24869 (13)	0.0531 (8)	
C4	0.4751 (3)	0.4373 (2)	0.22482 (12)	0.0499 (8)	
C5	0.4885 (3)	0.3360 (2)	0.18144 (11)	0.0422 (7)	
C6	0.3614 (2)	0.26764 (17)	0.16138 (10)	0.0338 (6)	
C7	−0.0609 (3)	0.3020 (3)	0.15644 (19)	0.0690 (10)	
C8	0.2429 (2)	0.09333 (19)	0.08520 (10)	0.0380 (6)	
C9	0.3815 (2)	0.16115 (17)	0.11258 (10)	0.0339 (6)	
C10	0.6581 (3)	0.01494 (19)	0.02078 (12)	0.0455 (7)	
C11	0.6974 (3)	−0.07862 (19)	−0.03287 (12)	0.0464 (7)	
C12	0.5872 (3)	−0.1500 (3)	−0.06694 (14)	0.0668 (10)	
C13	0.6332 (4)	−0.2383 (3)	−0.11655 (18)	0.0808 (13)	
C14	0.7839 (4)	−0.2554 (2)	−0.13190 (16)	0.0751 (12)	
C15	0.8968 (3)	−0.1863 (2)	−0.09916 (14)	0.0588 (8)	
C16	0.8497 (3)	−0.0975 (2)	−0.04930 (12)	0.0513 (8)	
C17	1.0632 (4)	−0.2042 (3)	−0.11637 (18)	0.0803 (11)	
H2	0.11120	0.43326	0.24642	0.0628*	
H3	0.32356	0.54297	0.27748	0.0637*	
H4	0.56141	0.48092	0.23799	0.0598*	
H5	0.58447	0.31247	0.16504	0.0506*	
H7A	−0.10558	0.32386	0.20337	0.1034*	
H7B	−0.12931	0.25007	0.12985	0.1034*	
H7C	−0.04256	0.37318	0.12755	0.1034*	
H8	0.27104	0.00984	0.07453	0.0456*	
H10	0.73577	0.06310	0.03951	0.0545*	
H12	0.48435	−0.13858	−0.05656	0.0802*	
H13	0.56053	−0.28641	−0.13963	0.0969*	
H14	0.81172	−0.31540	−0.16535	0.0899*	
H16	0.92277	−0.04956	−0.02640	0.0616*	
H17A	1.09454	−0.28229	−0.09956	0.1203*	
H17B	1.12213	−0.14373	−0.09134	0.1203*	
H17C	1.07907	−0.19801	−0.16917	0.1203*	

### Atomic displacement parameters (Å^2^)

**Table:**

	*U*^11^	*U*^22^	*U*^33^	*U*^12^	*U*^13^	*U*^23^
Cl1	0.0545 (4)	0.0921 (4)	0.0390 (3)	−0.0107 (3)	−0.0091 (3)	0.0114 (3)
S1	0.0304 (3)	0.0494 (3)	0.0434 (3)	−0.0036 (2)	0.0022 (2)	0.0065 (2)
O1	0.0360 (10)	0.0662 (10)	0.0655 (10)	−0.0139 (8)	−0.0005 (8)	0.0038 (8)
O2	0.0459 (10)	0.0616 (9)	0.0497 (8)	−0.0011 (8)	0.0023 (8)	0.0190 (7)
N1	0.0253 (10)	0.0544 (10)	0.0561 (11)	0.0053 (8)	0.0001 (8)	−0.0009 (8)
N2	0.0357 (10)	0.0437 (9)	0.0435 (9)	0.0038 (8)	0.0036 (8)	−0.0065 (7)
N3	0.0414 (12)	0.0459 (10)	0.0527 (10)	−0.0009 (9)	0.0088 (9)	−0.0098 (8)
C1	0.0322 (11)	0.0447 (11)	0.0374 (10)	0.0049 (9)	−0.0006 (8)	−0.0003 (8)
C2	0.0417 (13)	0.0592 (13)	0.0561 (13)	0.0133 (12)	0.0043 (11)	−0.0130 (12)
C3	0.0569 (17)	0.0487 (12)	0.0537 (12)	0.0045 (12)	−0.0028 (12)	−0.0115 (10)
C4	0.0488 (15)	0.0507 (12)	0.0501 (12)	−0.0065 (11)	−0.0107 (11)	−0.0038 (10)
C5	0.0331 (12)	0.0520 (12)	0.0414 (10)	−0.0031 (10)	0.0002 (9)	−0.0009 (9)
C6	0.0301 (11)	0.0399 (9)	0.0315 (9)	0.0022 (8)	−0.0007 (8)	0.0017 (8)
C7	0.0372 (15)	0.0779 (19)	0.092 (2)	0.0183 (12)	−0.0136 (14)	−0.0157 (17)
C8	0.0340 (11)	0.0412 (10)	0.0389 (10)	−0.0015 (9)	0.0017 (9)	0.0008 (9)
C9	0.0285 (11)	0.0386 (9)	0.0347 (9)	0.0022 (9)	0.0005 (8)	0.0035 (8)
C10	0.0416 (13)	0.0473 (12)	0.0475 (11)	0.0076 (10)	0.0017 (10)	−0.0071 (9)
C11	0.0489 (14)	0.0422 (11)	0.0481 (11)	0.0029 (11)	0.0066 (10)	−0.0052 (9)
C12	0.0661 (18)	0.0677 (16)	0.0667 (16)	−0.0120 (15)	0.0121 (14)	−0.0195 (14)
C13	0.093 (3)	0.0663 (17)	0.083 (2)	−0.0196 (17)	0.0097 (18)	−0.0307 (15)
C14	0.110 (3)	0.0500 (14)	0.0652 (16)	0.0004 (17)	0.0260 (17)	−0.0173 (12)
C15	0.0755 (18)	0.0493 (13)	0.0516 (12)	0.0193 (14)	0.0202 (14)	0.0017 (10)
C16	0.0553 (15)	0.0465 (12)	0.0521 (12)	0.0091 (12)	0.0076 (11)	−0.0046 (10)
C17	0.084 (2)	0.080 (2)	0.0770 (19)	0.0358 (17)	0.0277 (17)	0.0024 (16)

### Geometric parameters (Å, °)

**Table:**

Cl1—C8	1.7797 (19)	C12—C13	1.386 (4)
S1—O1	1.4237 (17)	C13—C14	1.364 (5)
S1—O2	1.4211 (16)	C14—C15	1.385 (4)
S1—N1	1.621 (2)	C15—C16	1.396 (3)
S1—C8	1.7763 (19)	C15—C17	1.505 (4)
N1—C1	1.428 (3)	C2—H2	0.9300
N1—C7	1.457 (3)	C3—H3	0.9300
N2—N3	1.411 (2)	C4—H4	0.9300
N2—C9	1.286 (2)	C5—H5	0.9300
N3—C10	1.269 (3)	C7—H7A	0.9600
C1—C2	1.387 (3)	C7—H7B	0.9600
C1—C6	1.400 (3)	C7—H7C	0.9600
C2—C3	1.374 (4)	C8—H8	0.9800
C3—C4	1.377 (4)	C10—H10	0.9300
C4—C5	1.375 (3)	C12—H12	0.9300
C5—C6	1.397 (3)	C13—H13	0.9300
C6—C9	1.484 (3)	C14—H14	0.9300
C8—C9	1.513 (3)	C16—H16	0.9300
C10—C11	1.459 (3)	C17—H17A	0.9600
C11—C12	1.393 (4)	C17—H17B	0.9600
C11—C16	1.384 (4)	C17—H17C	0.9600
			
O1—S1—O2	119.32 (10)	C16—C15—C17	120.8 (2)
O1—S1—N1	108.37 (10)	C11—C16—C15	122.0 (2)
O1—S1—C8	111.14 (9)	C1—C2—H2	119.00
O2—S1—N1	110.69 (10)	C3—C2—H2	119.00
O2—S1—C8	104.52 (9)	C2—C3—H3	120.00
N1—S1—C8	101.29 (10)	C4—C3—H3	120.00
S1—N1—C1	116.97 (14)	C3—C4—H4	120.00
S1—N1—C7	121.86 (17)	C5—C4—H4	120.00
C1—N1—C7	120.8 (2)	C4—C5—H5	119.00
N3—N2—C9	113.71 (16)	C6—C5—H5	119.00
N2—N3—C10	111.08 (17)	N1—C7—H7A	109.00
N1—C1—C2	118.8 (2)	N1—C7—H7B	110.00
N1—C1—C6	121.60 (17)	N1—C7—H7C	109.00
C2—C1—C6	119.6 (2)	H7A—C7—H7B	109.00
C1—C2—C3	121.1 (2)	H7A—C7—H7C	109.00
C2—C3—C4	119.9 (2)	H7B—C7—H7C	109.00
C3—C4—C5	119.7 (2)	Cl1—C8—H8	110.00
C4—C5—C6	121.6 (2)	S1—C8—H8	110.00
C1—C6—C5	118.04 (18)	C9—C8—H8	109.00
C1—C6—C9	122.46 (17)	N3—C10—H10	118.00
C5—C6—C9	119.47 (17)	C11—C10—H10	118.00
Cl1—C8—S1	108.93 (10)	C11—C12—H12	121.00
Cl1—C8—C9	110.45 (14)	C13—C12—H12	121.00
S1—C8—C9	108.89 (13)	C12—C13—H13	120.00
N2—C9—C6	118.41 (16)	C14—C13—H13	120.00
N2—C9—C8	121.94 (17)	C13—C14—H14	119.00
C6—C9—C8	119.63 (15)	C15—C14—H14	119.00
N3—C10—C11	123.1 (2)	C11—C16—H16	119.00
C10—C11—C12	122.2 (2)	C15—C16—H16	119.00
C10—C11—C16	118.5 (2)	C15—C17—H17A	109.00
C12—C11—C16	119.4 (2)	C15—C17—H17B	109.00
C11—C12—C13	119.0 (3)	C15—C17—H17C	109.00
C12—C13—C14	120.7 (3)	H17A—C17—H17B	109.00
C13—C14—C15	122.0 (3)	H17A—C17—H17C	109.00
C14—C15—C16	117.0 (2)	H17B—C17—H17C	109.00
C14—C15—C17	122.2 (2)		
			
O1—S1—N1—C1	−171.41 (15)	C1—C2—C3—C4	−0.3 (3)
O1—S1—N1—C7	1.7 (2)	C2—C3—C4—C5	0.9 (3)
O2—S1—N1—C1	55.99 (18)	C3—C4—C5—C6	−0.8 (3)
O2—S1—N1—C7	−130.9 (2)	C4—C5—C6—C1	0.1 (3)
C8—S1—N1—C1	−54.42 (17)	C4—C5—C6—C9	178.17 (19)
C8—S1—N1—C7	118.7 (2)	C1—C6—C9—N2	−178.30 (18)
O1—S1—C8—Cl1	49.97 (14)	C1—C6—C9—C8	3.4 (3)
O1—S1—C8—C9	170.49 (13)	C5—C6—C9—N2	3.7 (3)
O2—S1—C8—Cl1	179.94 (12)	C5—C6—C9—C8	−174.64 (17)
O2—S1—C8—C9	−59.54 (15)	Cl1—C8—C9—N2	−93.1 (2)
N1—S1—C8—Cl1	−64.97 (12)	Cl1—C8—C9—C6	85.20 (18)
N1—S1—C8—C9	55.54 (15)	S1—C8—C9—N2	147.38 (16)
S1—N1—C1—C2	−151.50 (17)	S1—C8—C9—C6	−34.4 (2)
S1—N1—C1—C6	28.4 (3)	N3—C10—C11—C12	3.8 (4)
C7—N1—C1—C2	35.3 (3)	N3—C10—C11—C16	−175.3 (2)
C7—N1—C1—C6	−144.8 (2)	C10—C11—C12—C13	−179.1 (2)
C9—N2—N3—C10	174.07 (18)	C16—C11—C12—C13	0.0 (4)
N3—N2—C9—C6	−176.04 (16)	C10—C11—C16—C15	179.1 (2)
N3—N2—C9—C8	2.2 (3)	C12—C11—C16—C15	0.0 (3)
N2—N3—C10—C11	−178.60 (19)	C11—C12—C13—C14	0.0 (4)
N1—C1—C2—C3	179.5 (2)	C12—C13—C14—C15	0.0 (5)
C6—C1—C2—C3	−0.4 (3)	C13—C14—C15—C16	0.0 (4)
N1—C1—C6—C5	−179.34 (18)	C13—C14—C15—C17	−179.5 (3)
N1—C1—C6—C9	2.6 (3)	C14—C15—C16—C11	0.0 (3)
C2—C1—C6—C5	0.5 (3)	C17—C15—C16—C11	179.6 (2)
C2—C1—C6—C9	−177.55 (19)		

### Hydrogen-bond geometry (Å, °)

**Table:**

*D*—H···*A*	*D*—H	H···*A*	*D*···*A*	*D*—H···*A*
C4—H4···O2^i^	0.93	2.57	3.469 (3)	163
C10—H10···O1^ii^	0.93	2.53	3.300 (3)	140

Symmetry codes: (i) −*x*+1, *y*+1/2, −*z*+1/2; (ii) *x*+1, *y*, *z*.

## References

[bb1] Bruker (2005). *SADABS* Bruker AXS Inc., Madison, Wisconsin, USA.

[bb2] Bruker (2009). *APEX2* and *SAINT* Bruker AXS Inc., Madison, Wisconsin, USA.

[bb3] Cremer, D. & Pople, J. A. (1975). *J. Am. Chem. Soc.* **97**, 1354–1358.

[bb4] Farrugia, L. J. (1997). *J. Appl. Cryst.* **30**, 565.

[bb5] Farrugia, L. J. (1999). *J. Appl. Cryst.* **32**, 837–838.

[bb6] Flack, H. D. (1983). *Acta Cryst.* **A**39, 876-881.

[bb7] Shafiq, M., Khan, I. U., Arshad, M. N. & Siddiqui, W. A. (2011*a*). *Asian J. Chem.* **23**, 2101–2106.

[bb8] Shafiq, M., Khan, I. U., Zia-ur-Rehman, M., Arshad, M. N. & Asiri, A. M. (2011*b*). *Acta Cryst.* E**67**, o2038. 10.1107/S1600536811027577 PMC3213487 22091066

[bb9] Shafiq, M., Khan, I. U., Zia-ur-Rehman, M., Arshad, M. N. & Asiri, A. M. (2011*c*). *Acta Cryst.* E**67**, o2092. 10.1107/S1600536811028406 PMC3213534 22091111

[bb10] Shafiq, M., Zia-ur-Rehman, M., Khan, I. U., Arshad, M. N. & Khan, S. A. (2011*d*). *J. Chil. Chem. Soc.* **56**, 527–531.

[bb11] Sheldrick, G. M. (2008). *Acta Cryst.* A**64**, 112–122. 10.1107/S0108767307043930 18156677

[bb12] Spek, A. L. (2009). *Acta Cryst.* D**65**, 148–155. 10.1107/S090744490804362X PMC2631630 19171970

